# Whole-Genome Sequencing of Multidrug-Resistant *Acinetobacter baumannii* Local Isolate and Molecular Dynamics Simulation Studies of a Modified KR-12 Analog Targeting AbaQ and BfmR

**DOI:** 10.3390/ijms27073107

**Published:** 2026-03-29

**Authors:** Farha Anwer, Sidra Anwar, Abdur Rahman, Amjad Ali, Abdul Rauf, Fazal Hanan, Mehvish Javeed

**Affiliations:** 1 Atta Ur Rahman School of Applied Biosciences (ASAB), National University of Sciences and Technology (NUST), Islamabad 44000, Pakistan; fanwar.phdabs17asab@student.nust.edu.pk (F.A.); sidraanwar957@gmail.com (S.A.); a.rahman@asab.nust.edu.pk (A.R.); 2 Medical Lab Technology (MLT), Faculty of Rehabilitation & Allied Health Sciences (FRAHS), Riphah International University, Al-Mizan Campus, Rawalpindi 46000, Pakistan; abdulrauf18335@gmail.com; 3 Saidu Group of Teaching Hospital, Saidu Medical College, Saidu Sharif 19200, Pakistan; drfhanan@gmail.com; 4 Pathology Department, Nishtar Hospital, Multan 60000, Pakistan; mehvishjaved15@yahoo.com

**Keywords:** *Acinetobacter baumannii*, antimicrobial peptides, multidrug resistance, molecular dynamics simulation

## Abstract

*Acinetobacter baumannii* (*A. baumannii*) represents a major threat because of its multidrug resistance, achieved through its ability to control virulence, and its mechanisms of drug efflux resistance. In this study, we used a combined experimental–computational approach to create and evaluate antimicrobial peptides that targeted the two essential pathogenic proteins, BfmR and AbaQ. The genomic analysis of a clinical isolate showed an extensive resistome and virulence profile, which matched high-risk global lineages. This study conducted molecular docking of an experimental AMP (cathelicidin KR-12 screened from the literature) and a rationally designed synthetic AMP (modified KR-12 analog) with pathogenic proteins, followed by 200 ns molecular dynamics simulations to evaluate both the binding stability and inhibitory potential of the compounds. The disk diffusion assay and microdilution assay were performed against *A. baumannii*. The study used comparative trajectory analyses, including RMSD, RMSF, radius of gyration, solvent-accessible surface area, principal component analysis, and MM-PBSA free energy calculations, to show that the synthetic AMP created stable electrostatic and hydrogen-bond networks, which caused conformational locking, and reached lower energy states than the experimental peptide. The synthetic AMP showed significant inhibition in validation in vitro. Contrastingly, the experimental AMP had transient interactions and no specificity. The study demonstrates that rationally designed AMPs have therapeutic potential, while the results create a reliable in silico framework to combat multidrug-resistant *A. baumannii*.

## 1. Introduction

*Acinetobacter baumannii* has become one of the most virulent pathogens of the 21st century [1]. The ESKAPE group (*Enterococcus faecium*, *Staphylococcus aureus*, *Klebsiella pneumoniae*, *Acinetobacter baumannii*, *Pseudomonas aeruginosa*, and *Enterobacter* species) serves as the main source of hospital-acquired infections, which include ventilator-associated pneumonia, bloodstream infections, and urinary tract infections [2]. The pathogen creates clinical challenges because it possesses exceptional capabilities to develop resistance against all forms of antibiotics, which now allows it to use extensively drug-resistant (XDR) and pan-drug-resistant (PDR) strains for global spread [3].

*A. baumannii* has multiple means of developing antibiotic resistance and causing disease. These bacteria achieve their resistance mechanisms through their use of beta-lactamase enzymes, which degrade drugs; their alteration of penicillin-binding proteins (PBPs) as essential target sites; and their production of multidrug efflux pumps at excessive levels [4]. The AbaQ pump serves as an important example because this pump provides protection against essential antibiotic groups, which include quinolones. The system has advanced control mechanisms, which include the BfmR network that governs the production of virulence factors, including biofilm formation—the main reason for treatment resistance in patients. The development of new inhibitors that successfully attack vital virulence factors, which range from pumps to complete control systems, stands as the most important research goal [5].

Researchers have found antimicrobial peptides (AMPs) to be an effective yet safe treatment option. The innate immune system uses AMPs as a defense mechanism that destroys bacterial cells through membrane destruction, while several AMPs can also attack bacterial targets inside the cell [6]. The advantages of AMPs include their broad-spectrum activity, rapid bacterial killing, and minimal risk of developing resistance [7]. The clinical application of this technology has faced obstacles because of two main problems, which are potential toxicity and unstable behavior in vivo.

Computational methods establish a strong framework that enables researchers to discover new and improve existing antimicrobial peptides. Rational design enables scientists to create new peptides through de novo design, which produces peptides with better charge characteristics, amphipathicity, and hydrophobicity [8]. Molecular docking can then screen these peptides against specific protein targets, which helps to determine their binding strength and mechanisms. Molecular dynamics (MD) simulations give scientists a detailed view of how peptides interact with their target proteins, which enables them to study complex stability and binding energy mechanisms [9].

KR-12 is an excellent choice for a therapeutic scaffold because of its sufficient efficacy, its safety, and its flexibility for sequence modifications. Being the smallest active fragment of human cathelicidin LL-37, KR-12 retains all important antimicrobial and immunomodulatory functions, but it has not been produced in large amounts, and it is not as degradable or cytotoxic as its parent molecule. It also has unique merits as compared to other peptide families. In contrast to the complex and expansive disulfide bond networks of defensins, KR-12 is a simple linear alpha-helix, and researchers can modify it structurally with ease. Moreover, clinical candidates such as pexiganan or fragments of LL-37 (e.g., FK-16) are larger and can be optimized to be used in membrane disruption or other activities, but KR-12 is the smallest, most effective structural core necessary to target bacterial regulators such as AbaQ and BfmR [10,11]. The AbaQ efflux pump (MFS family transporter) is a major contributor to active multidrug extrusion and is widely present in *A. baumannii*. The BfmR response regulator is a master controller of biofilm formation and global virulence. Having rationally designed a single peptide to act on both a direct resistance mechanism (AbaQ) and a core virulence/biofilm pathway (BfmR), we aimed to target two of the strongest defense mechanisms of the pathogen at once. It is believed that this dual-targeting strategy will contribute to reducing the likelihood of bacteria developing rapid mutational resistance [12,13].

Therefore, in this study, we conducted research to create a modified antimicrobial peptide, namely a synthetic KR-12 analog, from the KR-12 parent peptide, which we assessed for its ability to block the vital *A. baumannii* proteins AbaQ and BfmR. This study used molecular docking analysis combined with 200 ns MD simulations to discover a lead peptide and determine its binding mechanism through atomic-level analysis.

## 2. Results

### 2.1. Isolation and Identification of A. baumannii

**Figure 1:**
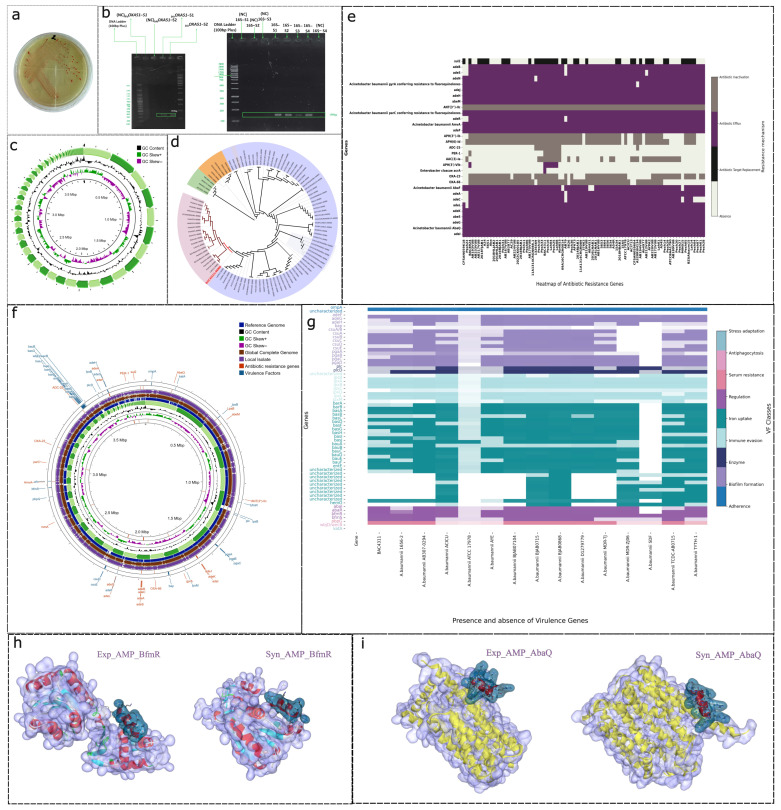
Genomic characterization and molecular docking of the *Acinetobacter baumannii* multidrug-resistant isolate. (**a**) Bacterial colony morphology on CHROMagar plate; (**b**) gel electrophoresis results indicating 16S rRNA and *blaOXA-51* gene sizes; (**c**) circular genome map displaying whole-genome view with GC content and skew; (**d**) phylogenetic tree indicating local isolate that was used in this study; background color (indigo, orange, and green) indicates various phylogenetic groups/clades; pink indicates shared clades; (**e**) heatmap of antibiotic gene resistance of different strains of *A. baumannii*; colors indicate whether particular resistance mechanisms are present (purple/grey) or absent (white); (**f**) comparative genomics analysis against reference genomes; (**g**) heatmap indicating presence and absence of virulence factors by class (indicated by color-coded legend on right); (**h**,**i**) molecular docking complexes of experimental (Exp_AMP) and synthetic (Syn_AMP) peptides with proteins BfmR (**h**) and AbaQ (**i**). The protein surfaces are depicted in transparent light purple and the secondary structures are displayed in ribbon format (red/yellow), while teal/dark red colors are used to highlight the peptides in the primary binding pockets.

We obtained *A. baumannii* from Nishtar Hospital Multan, Pakistan, and it was cultured on selective media, namely CHROMagar Acinetobacter; see Figure 1a. The successful isolation of *A. baumannii* was indicated by distinct red-pigmented colonies, a key indicator of *A. baumannii* on this medium. Polymerase chain reaction (PCR) was subsequently used for the molecular identification of *A. baumannii*; see Figure 1b. Amplification yielded two distinct bands: a 16S rRNA gene fragment of approximately 150 bp confirmed the presence of the Acinetobacter genus, and a 353 bp fragment corresponding to the *blaOXA-51* gene confirmed the species-level identification [14].

The clear identification of a 150 bp band suggests that these PCR-based detection methods are suitable for differentiating *A. baumannii* from other clinical pathogens at the genus level [15]. Furthermore, the presence of a clear band at 353 bp, which is characteristic of *A. baumannii*, offers clear confirmation of species-level identification [14].

### 2.2. Antimicrobial Susceptibility Testing for Phenotypic Assessment of Resistance Profile 

Phenotypic antimicrobial susceptibility testing indicated that the *blaOXA-23* and *blaOXA-51* beta-lactamase genes directly accounted for the isolate’s phenotypic resistance to carbapenems (imipenem and meropenem), cephalosporins (ceftriaxone and ceftazidime), and fluoroquinolones—specifically ciprofloxacin. The isolate was intermediately susceptible to levofloxacin, piperacillin, and tazobactam and susceptible to sulfamethoxazole and trimethoprim.

### 2.3. Genomic Analysis and Comparative Genomics

We utilized whole-genome sequencing methods to study the genetic structure of the local *A. baumannii* isolate. Quality checks indicated high read accuracy, and a high-quality genome was assembled (3.9 Mbp) and assessed in terms of completeness (99%). The GC content (38.96%) and presence of key genomic features are presented in the circular genome map in Figure 1c.

The phylogenetic comparison in Figure 1d depicts the isolate as part of a clade (highlighted in pink), mostly consisting of Pakistani isolates and the reference strain of *A. baumannii*. This clustering shows its genetic relatedness to previously reported pathogenic strains [16]. The clinical significance of this isolate is supported by its clustering with the other available *A. baumannii* isolates [17]. Thus, these findings suggest that the isolate may have similar mechanisms of resistance and pathogenic potential to these reported MDR strains.

### 2.4. Antimicrobial Resistance Gene Profiling

An extensive comparative genetic analysis shows multiple antimicrobial resistance gene determinants, as indicated in Figure 1e. This indicates the multidrug-resistant nature of this local isolate [17].

The identified components included efflux pump genes (*adeB*, *adeS*, *adeN*, *adeJ*, *adeH*, *abeM*, *adeK*, *adeG*, *adeL*, *adeA*, *adeC*, *AbaF*, *AbaQ*, *AmvA*, *abeS*), beta-lactamases (*OXA-23*, *OXA-66*, *ADC-25*, *PER-1*), aminoglycoside-modifying enzymes (*ANT(3″)-IIc*, *APH(3″)-Ib*, *APH(6)-Id*, *AAC(3)-Ia*, *APH(3′)-VIb*), fluoroquinolone resistance markers (*gyrA*, *parC*), and other resistance genes (*sul2*, *acrA*).

The increasing number of resistance determinants reflects a significant rise in antimicrobial resistance within *A. baumannii* [18]. Among all antibiotic resistance genes, those that encode efflux pumps play a significant role in reducing the effectiveness of current antibiotic medications, followed by beta-lactamase genes, which reflect resistance to widely used beta-lactam antimicrobials [19]. These findings are in accordance with previous reports that have highlighted *A. baumannii* as a priority pathogen that requires urgent antimicrobial intervention [18].

### 2.5. Virulence Factor Analysis

The virulence factor analysis revealed the existence of a number of adhesion, biofilm-forming, iron uptake, and immune evasion genes (Figure 1f,g). The presence of biofilm-associated genes such as *adeF-H*, *bap*, *csuA/B*, *csuA-E*, and *pgaA-D* also suggests the possibility of persistent infections and resistance to antimicrobial agents [20]. Moreover, genes such as *OmpA* were consistently present, noted for their roles in adhesion and host cell invasion [20]. Genes predominantly associated with iron uptake, such as *bauA*, *basD*, and *basE*, play critical roles in bacterial survival under iron-limited conditions [21]. Furthermore, immune evasion factors including *katA*, *lpxA*, *lpxB*, *lpxC*, *lpxD*, *lpxL*, *lpxM*, and *lpsB* were identified, which could serve as a means of countering host defenses [22].

Biofilm formation serves as a key factor in *A. baumannii*, offering protection against the host immune response and antibiotics. Meanwhile, regulatory genes, such as *BfmR*, are necessary for biofilm formation and antibiotic resistance and are hence important targets for further investigation [23]. The results show the clear importance of developing specific treatment strategies against multidrug-resistant infections.

### 2.6. Molecular Docking Studies of Antimicrobial Peptides

Molecular docking studies were conducted to analyze the binding of an experimental AMP (Exp_AMP) and a synthetic AMP (Syn_AMP) with two major pathogenic proteins of *A. baumannii*, BfmR and AbaQ. The binding interactions are shown in Figure 1h,i.

#### 2.6.1. BfmR Docking

- Exp_AMP_BfmR (experimental AMP): sixteen bonds formed (12 hydrophobic and 4 electrostatic).
- Synthetic AMP (Syn_AMP_BfmR): 21 bonds formed (3 salt bridges, 8 electrostatic interactions, 4 hydrophobic interactions, and six hydrogen bonds).

#### 2.6.2. AbaQ Docking

- Experimental AMP (Exp_AMP_AbaQ): eight bonds formed (2 hydrogen bonds and six hydrophobic interactions).
- Synthetic AMP (Syn_AMP_AbaQ): fifteen bonds formed (all hydrophobic).

In accordance with these findings, the synthetic AMP had more electrostatic and hydrogen bonds, which means that they had better stability and selectivity to bind to the target proteins. The potent interaction with BfmR identifies the synthetic AMP as an antimicrobial candidate.

Contrastingly, the Exp_AMP binding interaction with AbaQ was weak, comprising only six hydrophobic bonds, which indicates that its structure would need to be modified to enhance its efficacy. Overall, the study shows that synthetic AMPs can serve as an effective alternative to prevent *A. baumannii* infection by interfering with the major regulatory pathways [24].

### 2.7. Comparative Analysis of Molecular Dynamics and Binding Affinities of AbaQ-AMP Complex

**Figure 2:**
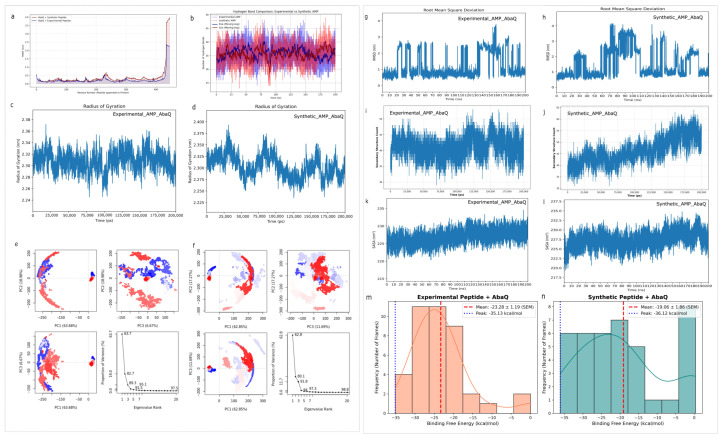
Schematic overview of comparison of AbaQ-AMP complexes. (**a**) RMSF for AMP-AbaQ complex, with blue color highlighting residue fluctuations, while residue fluctuations for synthetic complex are shown in maroon color. (**b**) Hydrogen-bonding plots across 200 ns molecular dynamics (MD) simulations for experimental (blue color) and synthetic AMPs (maroon color). (**c**,**d**) Radius of gyration comparison for assessment of compactness across 200 ns simulation. (**e**,**f**) PCA plots to analyze variance in PC1 and PC2 regions with eigenvalues. The corresponding eigen value (PCs) represents the percentage of overall mean square displacement of residue positional fluctuation in each dimension. The color value blue to white to red of periodic jump during 200 ns simulation. (**g**,**h**) RMSD used to analyze deviations to detect stable interactions. (**i**,**j**) DSSP for secondary structure predictions for stability. (**k**,**l**) SASA plots for surface-accessible area prediction. (**m**,**n**) Distribution of binding free energy, where vertical red dashed lines mark the average binding energy and blue dotted lines indicate the highest binding affinity across the entire simulation.

The comparative root mean square fluctuation (RMSF) profile in Figure 2a shows how peptide binding affects structural changes in the protein.

The experimental peptide depends on more localized interactions like PHE10 Pi-Alkyl contacts with LEU246 and ILE415, which result in elevated and fluctuating spikes around 230–240 and 350–370 residues.

The synthetic complex shows higher protein core stability (residues 350–370), and this micro-stability is supported by the interacting residues ILE411, ILE415, and ALA418, involved in a dense network of hydrophobic alkyl interactions. The protein establishes an induced fit mechanism through its structural changes, which result from the deep synthetic ligand insertion. This confirms that the synthetic peptide has a high affinity for specific targets, while the experimental AMP shows non-specific binding that lasts for a short time [25].

Hydrogen-Bond Dynamics Analysis: The binding patterns of the two peptides show different modes of bonding, as seen through the time evolution evaluation of hydrogen-bond formation, which is illustrated in Figure 2b. The experimental AMP exhibits strong and periodic fluctuations, decreasing to as few as 24 bonds at any given period; the synthetic AMP is more stable at a higher equilibrium, with a consistent range of 35 to 40 hydrogen bonds. This result is supported by the fact that the synthetic complex exhibits a highly repetitive and organized bonding pattern with AbaQ, involving residues like LYS419 and ILE411, which provide a consistent “anchor” for the peptide. Contrastingly, the experimental AMP depends on fewer specific bonding patterns with AbaQ, involving P:ARG6 (AMP) to A:TYR4 (AbaQ) as conventional hydrogen bonds, which may be more susceptible to the conformational shifting seen in the trajectory [26].

The backbone length and radius of gyration (*Rg*) confirm that the synthetic peptide causes a major conformational change; see Figure 2d. The synthetic complex starts from an active period of dynamic induced fitting (approximately 2.27 nm to 2.32 nm), which ends with protein movement into a more compact state (*Rg* ~ 1.88 nm), while the experimental complex maintains average compactness with sharp fluctuations, reaching up to 2.37 nm; see Figure 2c. The structural tightening process demonstrates that ILE411, ILE415, and ALA418 create a molecular anchor that protects the peptide inside the binding pocket [27].

A principal component analysis (PCA) was used to demonstrate the core system dynamics. The 2D projection of the first two eigenvectors shows a major difference between the two methods that they use to explore the conformational space. The experimental complex shows a continuous broad distribution across the phase space (Figure 2e), with PC1 variance of 63.68 percent. The protein maintains multiple structural states, while its red region trajectory frames show spatial distribution because it has not reached a single stable substate. The synthetic complex shows localized clustering in PC1 and PC2, with variance of about ~63 %, and faster convergence in the eigenvalue rank, with variance of 91.8%; see Figure 2f. The synthetic peptide induces a specific conformational change that causes the protein to enter a stable compact state according to the discovered clustering results and the radius of gyration and RMSF findings. The experimental complex remains conformationally dispersed because of non-specific binding, whereas the synthetic complex shows peptide-induced stabilization through tightly clustered subspace convergence, which leads to a well-defined bound state [28].

The root mean square deviation (RMSD) trajectory analysis shows that the synthetic ligand has better stability than the experimental AMP. The synthetic peptide maintains a low and stable deviation that remains constant at approximately 3.5 nm and 4.0 nm after the initial 80 ns throughout the entire 200 ns simulation; see Figure 2h. This shows that it maintains a stable binding pose. The experimental peptide (Figure 2g) shows sharp spikes at 0.5 nm and sudden jumps to 2.8 nm. The experimental AMP shows a significant loss of stability towards the end of the simulation (150–180 ns), peaking near 3.8 nm, which indicates that it cannot maintain a stable binding pose throughout the simulation process [29].

The secondary structure of proteins (DSSP) analysis shows that the mechanism of induced fit induces specific changes in protein conformation. The experimental complex (Figure 2i) maintains a static structural profile that shows structural changes through its residue movement between 35 and 45, rarely sustaining any significant structural improvement. The regular dips (with one even below 30 residues) are indicative of the temporary unfolding of secondary structure elements. The synthetic complex (Figure 2j) demonstrates increasing secondary structure development and reaches 50 to 65 residues throughout the entire simulation period. This increase establishes a direct relationship with the radius of gyration, which shows that the synthetic peptide causes protein compaction, while it helps to develop stable secondary structural elements that hold the target in a highly ordered inactive state [30].

The synthetic peptide shows a compacting effect, as seen in the solvent-accessible surface area (SASA) analysis. The synthetic complex (Figure 2l) stabilized at a lower average surface area of about 225–227 nm^2^, particularly between 20 and 120, than the experimental complex (Figure 2k), with a range of about 230–232 nm^2^. The protein’s tertiary structure shows more compact packing because the protein binds to the ligand, which causes hydrophobic elements to become hidden [31]. The observed SASA profile aligns perfectly with the radius of gyration data, confirming that the synthetic peptide drives the target protein into a more globular, stable, and closed conformation.

The MMPBSA trajectory for the synthetic complex shows a stabilizing trend through its negative slope, which is connected to the induced-fit compaction event, as shown in Figure 2n. The experimental peptide achieves a maximum average binding energy, which measures −23.28 kcal/mol, according to Figure 2m, but its binding trajectory shows unpredictable behavior because it does not contain the typical ‘locking’ phase with stabilized binding that occurs with the synthetic ligand. The peak affinity values determine the maximum interaction potential of each complex. The synthetic AMP had an extremely high maximum binding energy (−36.12 kcal/mol), which was compared with the experimental AMP (−35.13 kcal/mol), with the peak binding energy being more potent. Nevertheless, since transient states are represented by peak values, we systematically studied the thermodynamic distribution of the whole trajectory. The average binding free energy (Δ*G_bind_*) of the synthetic analog was estimated as −19.06 ± 1.86 kcal/mol (SEM), and it was similar to the experimental control (−23.28 ± 1.19 kcal/mol (SEM)). The frequency distribution histogram (Figure 2m,n) shows that the synthetic peptide is able to establish a steady baseline thermodynamic condition, as opposed to using only stochastic peak interactions. The 1 kcal/mol peak affinity difference has strong significance, because it shows that the synthetic peptide can reach better binding positions, which enable better molecular interactions through the hydrophobic bonding anchor than the experimental control can achieve. This pattern indicates that the synthetic complex achieves strong binding stability, which helps it to reach a more advantageous binding state compared to the unstable binding state that exists in the experimental complex [32]. The replicate is provided in Supplementary Figure S1.

### 2.8. Comparative Analysis of Molecular Dynamics and Binding Affinities of BfmR-AMP Complex

**Figure 3:**
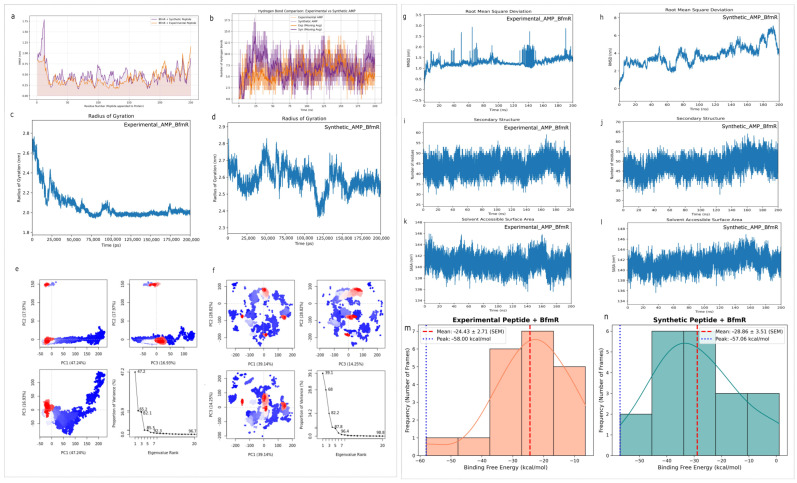
BfmR-AMP complexes simulated by comprehensive molecular dynamics (MD) over 200 ns. (**a**) RMSF, where shadowed regions show standard deviation (SD) values and indicate the trajectory’s movement pattern, while solid lines show the trajectory’s average movement. (**b**) Shadowed regions show statistical variance in hydrogen-bond evolution, while solid lines display the average number of persistent hydrogen-bond interactions. (**c**,**d**) Radius of gyration (*Rg*) comparative analysis reflecting structural compactness. (**e**,**f**) Principal component analysis (PCA) plots representing conformational variance; red areas indicate the most frequent conformational states (highest density), while blue areas indicate transition states. (**g**,**h**) RMSD plots depicting structural stability and deviations. (**i**,**j**) DSSP analysis for secondary structure transitions. (**k**,**l**) Solvent-accessible surface area (SASA) predictions. (**m**,**n**) Binding free energy distribution; vertical red dashed lines indicate the mean binding energy, while blue dotted lines represent the peak binding affinity observed during the simulation.

The RMSF analysis measured the protein backbone and peptide segments’ flexibility during the simulation experiments, as shown in Figure 3a.

Synthetic Complex: The protein backbone from residues 25 to 240 maintained a highly rigid profile that showed fluctuations that remained below 0.50 nm. The presence of high-energy salt bridges creates a connection between two P:ARG6 and P:ARG2 points, which connect to A:ASP90 at distances of 2.67 Angstroms and 3.05 Angstroms and to A:ASP103 at a distance of 4.18 Angstroms. Experimental Complex: The N-terminus showed initial stability, while the protein core and C-terminal regions showed greater residual flexibility. The experimental peptide establishes a primary electrostatic connection with A:ASP131 through long-range attraction, which measures 5.38 Angstroms, and fails to create a binding pocket that can maintain its shape [33].

The hydrogen-bond occupancy was measured to track the binding interface persistence across a 200 ns simulation; see Figure 3b. The synthetic AMP peptide showed superior H-bond performance because the peptide maintained a moving average of 10 to 12 bonds, while achieving a maximum value of eighteen bonds. The docking pose established strong conventional H-bonds, which supported the dynamic results through the identification of a P:ARG6 and A:THR88 interaction at 2.26 Angstroms. The synthetic AMP exhibited a significantly stronger and more consistent interaction profile, which was indicated by the fact that its hydrogen bond (H-bond) count was consistently higher, and its interactions sometimes attained higher levels, with 18 bonds [34]. The experimental system operated at a much lower baseline, which showed normal changes between four and seven H-bonds. The bonding trajectory experienced frequent losses because the system used non-directional hydrophobic Pi-alkyl interactions with A:TRP169 at 5.42 Angstroms, which created no directional bonds.

The radius of gyration and solvent-accessible surface area were used to measure the complex folding stability and compactness. The *Rg* for the synthetic AMP fluctuated between 2.4 nm and 2.8 nm, while maintaining a 2.6 nm average, indicating stable behavior, as shown in Figure 3d. The SASA profile shows that the synthetic peptide maintains the functional structural volume of the BfmR protein while protecting its hydrophobic active site from solvent contact, as shown in Figure 3l.

The experimental AMP showed a dramatic structural trend, which started with *Rg* at 2.75 nm and ended with *Rg* at approximately 2.0 nm during the simulation process, according to Figure 3c. The significant contraction, which the SASA showed as a downward trend, indicated that the binding pocket structure had collapsed or excessively contracted because the peptide’s weak interaction footprint became unstable, as seen in Figure 3k.

Overall, the synthetic complex maintained its complete structural integrity, which allowed active-site access, while the experimental complex showed over-compaction and reduced solvent exposure, which indicate binding-induced structural instability [35,36].

The two complexes were subjected to PCA, which enables researchers to study conformational patterns and vital movement patterns. The synthetic AMP’s PCA plot shows multiple red clusters that occupy small areas, while showing a high density because the system spends a large amount of time in one stable conformational state. The ASP90 regulatory domain of the protein maintains its inhibitory position because the electrostatic lock forcefully stops all its global movements. The experimental AMP’s PCA profile shows multiple separate areas of distribution, which create distinct islands across the entire experimental area. The distribution pattern shows that the peptide continuously changes its shape because it cannot maintain one stable position due to its weak binding to peripheral elements, which include A:ALA134 and A:ARG187.

An evaluation of structural equilibrium and time-dependent stability was conducted through the measurement of the RMSD, which was assessed through the BfmR-AMP complex. The synthetic AMP complex system acquired structural equilibrium quickly, where the protein backbone stabilized at 0.8 nm to 1.0 nm; see Figure 3h. The synthetic AMP maintained a consistent trajectory at 2 nm to 4.0 nm and a low and stable deviation after the first 80 ns. The system maintained its stability because its short-range H-bonds and salt bridges, which operate within 3.0 Angstroms, enable it to maintain high residence times [27]. The experimental AMP system displayed extreme volatility, where the RMSD showed multiple sharp spikes that reached 3.0 nanometers, as shown in Figure 3g. The transitions demonstrate flickering binding, since long-range electrostatic and hydrophobic forces that extended to 3 Angstroms failed to stop the peptide from moving or partially unbinding during thermal fluctuations.

The analysis used DSSP to monitor secondary structure elements because it was necessary to assess alpha-helices and beta-sheets throughout the entire trajectory. The secondary structure of the synthetic AMP system showed high stability because both alpha-helices and beta-sheet core elements maintained their original forms, as shown in Figure 3j. The protein maintained its native structure because salt bridge connections created a high binding density, which stopped all unfolding processes. The experimental AMP system demonstrated major structural loss because helical sections were transformed into both unstructured coils and turns, which was in line with the collapsing *Rg* shown in Figure 3i. The experimental peptide showed structural weakness through its C-terminus unfolding, which demonstrated that it could not maintain the protein’s structural integrity.

The synthetic peptide demonstrates superior performance, as binding free energy calculations through thermodynamic affinity measurements provided conclusive evidence of its advantages. The experimental AMP reached the highest peak binding energy of −58.00 kcal/mol against the BfmR target, which was highly similar to the peak of the synthetic peptide of −57.06 kcal/mol. To statistically evaluate this energetic profile with time, the average binding free energy was estimated. It is worth noting that the synthetic AMP had a significantly better and more stable average thermodynamic profile (−28.86 ± 3.51 kcal/mol SEM) compared to the less stable distribution of the experimental peptide (−24.43 ± 2.71 kcal/mol SEM). The synthetic modifications, as evidenced by the distribution histograms (Figure 3m,n), managed to stabilize the peptide in a high-affinity bound state, which was maintained consistently, reducing the transient fluctuations that were experienced in the control.

### 2.9. In Vitro Antimicrobial Activity of Synthesized AMPs

**Figure 4:**
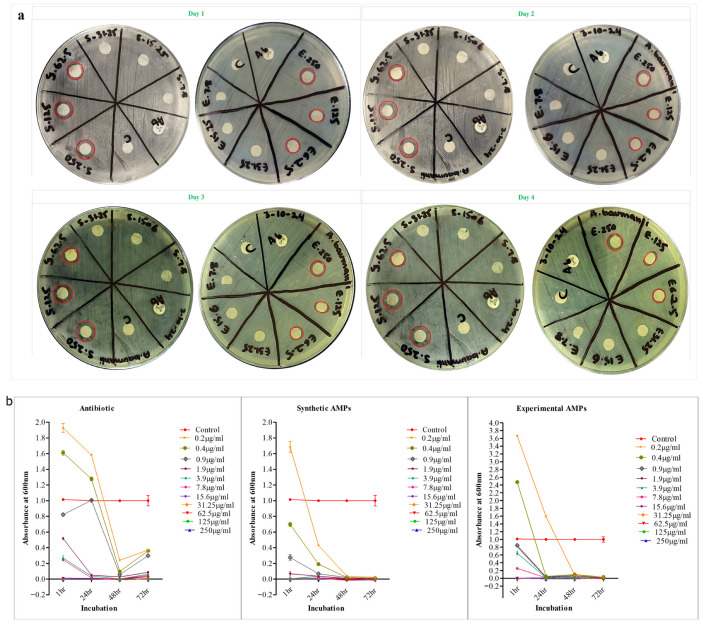
In vitro assays to confirm antimicrobial activity against *A. baumannii* local isolate. (**a**) Disk diffusion assay showing clear inhibition zones with red circles over 4 days of incubation; (**b**) minimum inhibitory concentrations from microdilution assay, showing the trends in antimicrobial activity up to 72 h of incubation.

The antimicrobial activity of the synthetic (S) and experimental (E) KR-12 cathelicidin was assessed against *A. baumannii* over four days, using concentrations from 250 µg/mL to 0.2 µg/mL. As seen in Figure 4a, significant zones of inhibition were observed for both AMP variants, with the experimental AMP showing consistently larger zones compared to the synthetic version. Ciprofloxacin, the positive control, exhibited the highest activity, but the experimental AMP demonstrated comparable results at higher concentrations (250 µg/mL).

A four-day duration was chosen to evaluate peptide stability and sustained efficacy, as AMPs can degrade over time due to proteases or oxidation [37]. Prolonged exposure also reflects real clinical conditions and helps to assess bacterial adaptive responses, such as biofilm formation or efflux-mediated resistance [38].

The synthetic KR-12 showed stronger and more sustained inhibition than the experimental version due to better stability and purity [37]. The decline in activity over time may indicate peptide degradation or resistance mechanisms, emphasizing the need for AMP optimization [39].

### 2.10. Broth Microdilution Assay

Broth microdilution assays (Figure 4b) showed that the experimental AMP achieved an MIC ≥ 3.9 μg/mL after 24–72 h. Lower concentrations (≤1.9 μg/mL) showed incomplete inhibition and regrowth after 24 h. Synthetic AMPs showed strong antimicrobial activity and achieved an MIC of 0.4 g/mL, even showing inhibition of 0.2 g/mL. In contrast, the experimental AMP showed less efficacy as little to no inhibitory effect was found at either concentration. The experimental cathelicidin KR-12 was confirmed to be effective against multidrug-resistant *A. baumannii* [40], with an MIC (≥125 µg/mL, 24 h). Its synthetic variant, however, engineered with amino acid substitutions, showed rapid and potent inhibition (MIC ≥ 31.25 µg/mL after 18 h). These results highlight the importance of incubation to achieve the maximum effect that KR-12 is capable of, as well as the effect that structural optimization has in overcoming bacterial resistance [41]. This suggests rational design as a powerful strategy to bridge the gap in the efficacy of natural and synthetic peptides.

## 3. Discussion

This research study provides an experimental and computational analysis of a multidrug-resistant *A. baumannii* clinical isolate, which was confirmed through selective culturing and PCR-based 16S rRNA and intrinsic *blaOXA-51* gene detection [42]. Whole-genome sequencing, together with phylogenetic analysis, showed that the isolate belongs to a clade that includes Pakistani and reference MDR strains, because they share both evolutionary and pathogenic characteristics. The extensive resistome, which contains multiple efflux pump systems and β-lactamases, together with the diverse virulome, which includes biofilm-associated and iron acquisition and immune evasion genes, demonstrates the clinical threat that this isolate poses and establishes the need for new treatment methods that do not use traditional antibiotics [43].

The synthetic AMP showed better performance than the experimental peptide when testing both AbaQ and BfmR through molecular docking and dynamic simulations that focused on crucial regulatory proteins together with resistance-related proteins. The synthetic AMP established permanent electrostatic connections through hydrogen bonding and salt bridge formation, which resulted in induced-fit binding that decreased protein structural movements while creating distinct conformational groups [44]. The experimental AMP showed unstable binding through its weak non-specific hydrophobic interactions, which caused surface-sliding behavior and produced high RMSD and RMSF values, together with scattered conformational states [45,46]. RMSD and *Rg* graphs showed the stability and convergence of our simulations when the two graphs were horizontalized after the first stage and became steady. The fact that the target–ligand complexes achieved a stabilized and balanced condition without creaming over time is evidence of this flat plateau. Moreover, to enable others to reproduce our work, we have clearly described all computer settings, force fields, and steps in the Section 4.

In the thermodynamic analysis, it was shown that the synthetic AMP achieved better and longer-lasting binding free energy results than the experimental peptide, which displayed unpredictable binding behavior despite showing occasional peaks in affinity. Contrastingly, it was observed that the standard commercial efflux pump inhibitor PAβN has a binding free energy of about −4.54 kcal/mol with *A. baumannii* efflux transporters. By this standard, our rationally designed synthetic peptide (−29.0 kcal/mol) is shown to exhibit a better and highly competitive binding energy profile [47]. The synthetic AMP showed effectiveness against the global BfmR regulator in *A. baumannii* because it disrupted biofilm formation and virulence, while also decreasing antimicrobial resistance [23]. A recent study has found that small-molecule BfmR inhibitors have a binding energy of approximately −25.3 kcal/mol. Comparatively, the maximum peak binding energy of the synthetic peptide was much higher at −57.06 kcal/mol. Consequently, the sequence modification used to create the synthetic AMP led to stronger peak binding energies, showing that it could be a promising candidate against *A. baumannii* [48].

The correlation between the binding energy, radius of gyration, RMSF, and PCA revealed the inhibition mechanism of the synthetic peptide. By restricting large-scale conformational locking, synthetic peptides limit pathogenic proteins to undergo induced-fit modeling [49].

Although our *in silico* predictions are well supported by the recently performed in vitro antimicrobial testing (disk diffusion and microdilution assays), we acknowledge the limitations of these results regarding clinical outcomes. The step between in vitro and in vivo activity is complicated. The experimental systems that are currently in use are not entirely reflective of the physiological challenges in the real world, including the possible proteolytic breakdown of the peptides in human serum, exogenous mammalian cytotoxicity, or the physical obstacles posed by an established infection in a living host. Thus, although our joint computational and in vitro results strongly substantiate the notion that the synthetic KR-12 analog is a powerful antibacterial agent, such results are preliminary. In the future, in vivo studies in mouse models and pharmacokinetic profiling are needed to confirm its real therapeutic potential and clinical significance.

## 4. Materials and Methods

**Figure 5:**
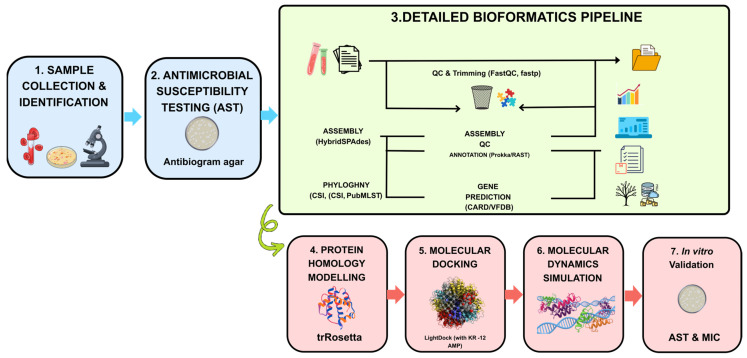
Overall methodology. Schematic representation of the overall computational workflow employed in this study, divided into 7 steps: (1) sample collection and identification; (2) antimicrobial susceptibility testing (AST); (3) detailed bioinformatics pipeline; (4) protein homology modeling; (5) molecular docking; (6) molecular dynamics simulation; and (7) in vitro validation. The blue and red arrows indicate the order of the workflow. The light green curly connector signifies that the gene prediction outcomes (CARD/VFDB) of the bioinformatics pipeline are integrated into the protein homology modeling to select the target for docking. Each stage is defined with software and database tools (e.g., FastQC, Prokka, TrRosetta, LightDock).

The methodology overview is presented in Figure 5.

### 4.1. Collection, Isolation, Characterization, and Identification of Bacterial Samples

Bacterial samples isolated from sputum were collected with charcoal swabs (Oxoid Ltd., Basingstoke, UK) from Nishtar Hospital, Multan, Pakistan. The bacterial samples were then isolated on CHROMagar Acinetobacter (CHROMagar, Paris, France), followed by overnight incubation at 37 °C. Gram staining and biochemical characterization were also performed—specifically, catalase and oxidase tests for the identification of *A. baumannii*. High-quality genomic DNA was extracted using a Bacterial Genomic DNA Extraction Kit (Solarbio Science & Technology Co., Ltd., Beijing, China), as per the manufacturer’s protocol. Gradient PCR was performed via the T100™ Thermal Cycler (Bio-Rad Laboratories, Inc., Hercules, CA, USA) to identify *Acinetobacter* spp., targeting the 16sRNA and *blaOXA-51* genes.

### 4.2. Antibiotic Susceptibility Testing by Disk Diffusion Method

The antimicrobial susceptibility profile was determined by the Kirby-Bauer disk diffusion assay using a panel of commercially available antibiotic disks (Oxoid Ltd., Basingstoke, UK), and the inhibition zones indicating susceptibility, intermediate susceptibility, and resistance were determined using the latest Clinical Laboratory Standards Institute (CLSI) standards [50].

### 4.3. Whole-Genome Sequencing, Comparative Analysis, and Molecular Docking

The genomic DNA was extracted using the abovementioned kit for bacterial genome extraction. The integrity of the DNA was assessed by gel electrophoresis (Bio-Rad, Hercules, CA, USA) and quantification was performed with a NanoDrop™ 2000 spectrophotometer (Thermo Fisher Scientific, Waltham, MA, USA). The whole-genome sequencing of the strain was performed on the DNBseq™ sequencing platform (MGI Tech Co., Ltd., Shenzhen, China) with a short-insert library by BGI Genomics, Shenzhen, China. The complete genomic sequence of this clinical isolate was submitted to NCBI GenBank and is publicly accessible under the accession number JBVTMM010000000. The quality of the FASTQ paired-end reads was assessed using FastQC v0.11.9 (https://www.bioinformatics.babraham.ac.uk/projects/fastqc/ (accessed on 5 November 2025)) [51]. The removal of adapter sequences and contamination was performed via the fastp tool v0.12.4 [52] to achieve base calling with Phredscore Q30 or above. Genome assembly was achieved using HybridSPAdes v3.15.3 [53], and the quality assessment of the genome assembly was conducted using the QUAST tool v4.4 [54]. Structural and functional genome annotations were performed utilizing Prokka v1.14.5 [55] and the RAST tool [56] (https://rast.nmpdr.org/rast.cgi (accessed on 5 November 2025)), respectively.

The genome assemblies of *A. baumannii* (*n* = 99), including 28 local draft and 71 global complete genomes, were retrieved from the PATRIC database (https://www.bv-brc.org/ (accessed on 11 November 2025)) [57]. The whole-genome SNP phylogeny was constructed via CSI Phylogeny 1.4 (https://cge.food.dtu.dk/services/CSIPhylogeny/ (accessed on 14 November 2025)) [58]. The phylogenomic tree was visualized by iTOL (https://itol.embl.de/personal_page.cgi (accessed on 15 November 2025)). The whole genome of the isolate of *A. baumannii* was visualized using Proksee (https://proksee.ca/ (accessed on 5 November 2025)) [59]. cgMLST was performed using the PubMLST database (https://pubmlst.org/ (accessed on 15 November 2025)) [60]. The genome assemblies of *A. baumannii* (*n* = 99) were used to identify antibiotic resistance genes from the Comprehensive Antibiotic Resistance Database (CARD) Resistance Gene Identifier (RGI) (https://github.com/arpcard/rgi (accessed on 17 November 2025)) [61]. Virulence factors of the isolate were predicted via the Virulence Factor Database (VFDB) (http://www.mgc.ac.cn/VFs/ (accessed on 17 November 2025)) [62].

### 4.4. Rational Peptide Design

The mapping of the exact sequence modifications was performed to guarantee computational reproducibility and clarity regarding the structure of the peptide. The wild-type parent KR-12 peptide has a 12-amino-acid sequence of KRIVQRIKDFLR. Four amino acid analog substitutions were rationally designed in silico to produce the synthetic analog sequence. The precise replacements that were made were valine to cysteine at position 4 (V4C), aspartic acid to cysteine at position 9 (D9C), phenylalanine to lysine at position 10 (F10K), and leucine to isoleucine at position 11 (L11I).

### 4.5. Molecular Docking

The protein sequences from CARD and VFDB were homology-modeled with trRosetta (https://yanglab.qd.sdu.edu.cn/trRosetta/ (accessed on 20 November 2025)) [63], and their molecular docking interactions were studied using LightDock (https://github.com/lightdock/lightdock (accessed on 21 December 2025)) [64] with the top-ranking KR-12 cathelicidin (experimental and synthetic) AMP from our previously published database [65].

### 4.6. Molecular Dynamics Simulation

A 200 ns simulation was performed using GROMACS 2024.04 (https://www.gromacs.org/ (accessed on 10 January 2026)) for each system, which included *Acinetobacter baumannii* pathogenic protein complexes with two peptides: an experimental AMP that represented the unmodified parent peptide retrieved from the literature and a synthetic AMP that was a rationally modified form with specific amino acid substitutions. The CHARMM36 all-atom force field was used to define protein atoms in the study. CGenFF-compatible parameters were used to define ligands. The system centered each complex in a cubic periodic box that required a minimum 1.0 nm distance from all solute atoms to the box edges. The CHARMM-modified TIP3P (TIPS3P) water was used to create a solvated environment for the experiments, which included NaCl ions that were used to neutralize the total charge while achieving 150 mM ionic strength in required instances.

The steepest descent algorithm achieved energy minimization until the force value reached 1000 kJ mol^−1^ nm^−1^. The maximum number of steps was 50,000, with a starting step size of 0.01 nm. High convergence was achieved by updating the neighbor list at each step, and cutoffs of 1.2 nm were used for both Van der Waals (force switch) and electrostatic (PME) interactions. The system underwent two equilibration phases, which included positional restraints for heavy atoms during NVT and NPT simulations. The NVT simulation lasted 1.0 ns at 300 K with the velocity-rescale thermostat, which had a time constant of 0.1 ps. The NPT simulation lasted 1.0 ns at 1 bar with the Parrinello–Rahman barostat, which had a time constant of 2.0 ps and isotropic coupling. The production MD simulations ran for 200 ns with a 2 fs time step while using the md integrator, Verlet neighbor lists, and PME electrostatics, as well as a 1.0 nm Lennard–Jones cutoff with long-range dispersion correction and LINCS constraints on hydrogen atom bonds. The system used periodic boundary conditions in all directions, while it recorded trajectories every 10 ps.

The trajectory analyses used RMSD, RMSF, SASA, radius of gyration, per-residue hydrogen bonding, protein–ligand interaction fingerprints, MM-GBSA binding free energy calculations, and collective motion analyses, combined with DCCM dynamic cross-correlation matrices and PCA with Cα covariance.

### 4.7. Antimicrobial Susceptibility of Synthesized AMPs

The working solution of KR-12 cathelicidin (experimental and synthetic) was created by weighing 0.25 mg of the lyophilized peptide (Peptide 2.0, Inc., Chantilly, VA, USA) with 1 mL of 0.1% DMSO to give a stock solution of 250 μg/mL. Thereafter, serial dilutions of the compound were created with 0.1% dimethyl sulfoxide (DMSO; Sigma-Aldrich, St. Louis, MO, USA), resulting in working concentrations of 250 µg/mL–0.2 µg/mL. The evaluation of the antimicrobial activity of the AMPs was performed using the disk diffusion method on Mueller–Hinton agar (MHA) (Oxoid, Basingstoke, UK) using the CLSI guidelines [50], utilizing sterile paper discs dissolved in dilutions 1 h before the experiment. Each assay was performed in triplicate with the positive control (ciprofloxacin) and negative control (DMSO only), plates were incubated at 37 °C, and zones of inhibition were observed and recorded daily for four days.

### 4.8. Broth Microdilution

The second method involved broth microdilution tests in 96-well plates (Greiner Bio-One, Kremsmünster, Austria) using phosphate-buffered saline (PBS; Thermo Fisher Scientific, Waltham, MA, USA). In MHB, the bacterial culture reached 10^6^ CFU/mL and then underwent centrifugation before re-suspending with PBS. Each well contained 10 µL of AMP solution, antibiotics, and a fraction with 90 µL of the bacterial suspension. The plate underwent incubation at 37 °C for 1 and 24 h. Measurements of absorbance took place at 1 h, 24 h, 48 h, and 72 h at a 600 nm wavelength using a microplate reader (Multiskan™ GO Microplate Reader (Thermo Fisher Scientific, Waltham, MA, USA)). The methodology was adopted from the previous literature, with minor adjustments [66,67,68].

## 5. Conclusions

The research findings demonstrate that synthetic AMPs created through rational design can achieve better performance than peptides discovered through experimental work or natural sources, because synthetic AMPs can establish permanent links to critical regulatory proteins. These results provide compelling in silico evidence that rationally designed AMPs with amino acid substitutions can enhance target specificity, stability, and inhibitory efficacy. The given synthetic AMP is a suitable candidate to be experimentally validated and optimized. The present work provides a model computational pipeline that can be used to speed up the discovery of next-generation antimicrobial peptides against multidrug-resistant pathogens.

The study demonstrates that the whole-genome sequencing of a local *A. baumannii* isolate enables the retrieval of target pathogenic proteins from a local isolate, producing better biological results and more practical research outcomes than when using existing protein databases. The isolate-specific modeling method enables scientists to identify genetic differences between strains that affect how peptides bind to proteins and thus enables more precise docking predictions, authentic molecular dynamics patterns, and distinct identification. The development of new antimicrobial drugs should focus on using whole-genome isolates because they enable more effective antimicrobial drug development against multidrug-resistant pathogens.

## Data Availability

The genomic sequence data generated for this study were deposited in the NCBI GenBank database under BioProject PRJNA1431334 (https://www.ncbi.nlm.nih.gov/bioproject/PRJNA1431334/ (accessed on 4 March 2026)) and GenBank accession number JBVTMM000000000. Data from the in silico molecular dynamics simulations, including the system setup and representative trajectories, and in vitro assays are available from the corresponding author upon reasonable request.

## Supplementary Materials

The following supporting information can be downloaded at https://www.mdpi.com/article/10.3390/ijms27073107/s1.

## Abbreviations

The following abbreviations are used in this manuscript:

XDR	Extensive Drug-Resistant
PDR	Pan Drug-Resistant
PBPs	Penicillin-Binding Proteins
AMPs	Antimicrobial Peptides
MD	Molecular Dynamics
RMSF	Root Mean Square Fluctuation
Rg	Radius of Gyration
PCA	Principal Component Analysis
RMSD	Root Mean Square Deviation
DSSP	Define Secondary Structure of Proteins
SASA	Solvent-Accessible Surface Area
