# Supplementary material for: Whole-Genome Sequencing of Multidrug-Resistant Acinetobacter baumannii Local Isolate and Molecular Dynamics Simulation Studies of a Modified KR-12 Analog Targeting AbaQ and BfmR
Source: Int J Mol Sci. 2026 Mar 29;27(7):3107. doi: 10.3390/ijms27073107 (PMC13073944; doi:10.3390/ijms27073107)
Supplement: Supplementary file 1 [file ijms-27-03107-s001.zip › ijms-4193356-supplementary.pdf]

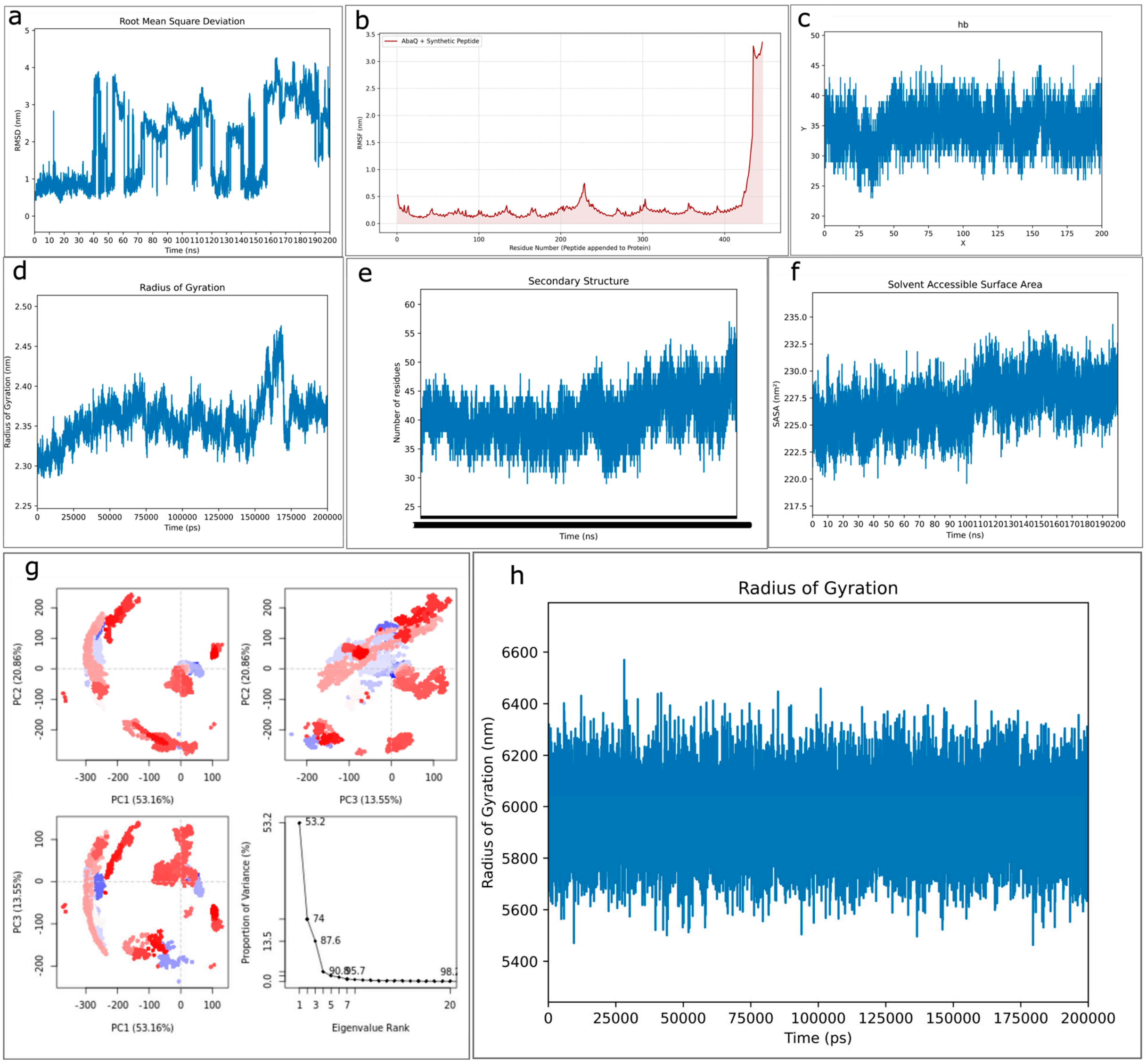

Figure S1: Molecular Dynamics track of analysis of the AbaQ-synthetic peptide complex (200ns). (a) Root mean square deviation (RMSD) of syn-AbaQ shows stability and less deviation; (b) Root mean square fluctuation (RMSF) shows less residues fluctuation and stable core; (c) Stable hydrogen bond formation through entire simulation; (d,h) Radius of Gyration (*Rg*) shows compactness of the complex; (e) secondary structure analysis shows high structural stability and less confirmational changes; (f) syn-AbaQ complex shows stable and lower Solvent accessible surface area (SASA) profile till 100 ns; (g) PCA analysis shows the synthetic complex is more restricted in conformational subspace. These findings indicate a very high reproducibility rate as compared to previous experiments demonstrating the stability of the simulation setting and structural integrity of the AbaQ-peptide binding interface.
